# Molecular subtyping of Alzheimer's disease using balanced integrative multi‐omics, deep learning, and multi‐scale network models

**DOI:** 10.1002/alz70855_103464

**Published:** 2025-12-23

**Authors:** Yacoub Abelard Njipouombe Nsangou, Maria A. Wörheide, Jürgen Dönitz, Gabi Kastenmuller, Matthias Arnold

**Affiliations:** ^1^ Helmholtz Zentrum München ‐ German Research Center for Environmental Health, Neuherberg, Germany; ^2^ Universitätsmedizin Göttingen, Göttingen, Germany; ^3^ Duke University, Durham, NC, USA; ^4^ Helmholtz Zentrum München ‐ German Research Center for Environmental Health, Neuherberg, 85764, Germany

## Abstract

**Background:**

Alzheimer's disease (AD) is a complex neurodegenerative disorder that manifests differently across patients, complicating both accurate diagnosis and development of personalized treatments. Advances in biotechnology, particularly omics technologies like transcriptomics, metabolomics, and proteomics, now allow researchers to gather comprehensive molecular data about the disease. The complementarity of information across omics types can not only improve our understanding of disease mechanisms but also help us identify distinct patient subgroups to enable personalized medicine approaches.

**Method:**

We here propose a multi‐omics data integration and disease subtyping approach leveraging data‐driven multi‐scale network structures from the AD Atlas. To this end, we first extract representative network module features from brain transcriptomics, proteomics, and metabolomics data from 356 ROS/MAP participants using Weighted Gene Co‐expression Network Analysis (WGCNA) and Principal Component Analysis (PCA) to balance omics feature set sizes. Second, we use deep graph representation learning on the multi‐scale AD Atlas networks followed by unsupervised clustering to obtain 25 AD‐relevant, functional multi‐omics clusters. Finally, we employ an autoencoder model to learn an integrated multi‐omics expression profile for each of these clusters based on representative omics features. This integrated expression matrix is then used for disease subtyping.

**Result:**

Our findings demonstrate that the autoencoder model is very effective in learning a low‐dimensional representation of multi‐omics features using balanced feature set sizes and predefined but data‐driven multi‐scale network clusters. As we show, this integrated multi‐omics expression matrix facilitates the identification of molecular subtypes of AD that might not be evident through more traditional analysis. On the phenotypic level, we identify the brain multi‐ome to be sensitive to both AD neuropathologies and clinical/cognitive status, as well as other pathological events and demographic/socioeconomic variables.

**Conclusion:**

The proposed multi‐omics integration methodology provides a robust framework for patient stratification and disease subtyping in AD. By integrating transcriptomics, proteomics, and metabolomics data with the multi‐scale AD atlas network, we identify patient groups with shared disease‐relevant clinical parameters, advancing our understanding of the molecular heterogeneity and respective subtype‐specific manifestations of Alzheimer's disease.

